# GQ-DNABERT reveals GQ proximal enhancer–promoter interactions associated with tissue-specific transcription

**DOI:** 10.1093/nar/gkaf1007

**Published:** 2025-10-14

**Authors:** Dmitry Konovalov, Dmitry Umerenkov, Alan Herbert, Maria Poptsova

**Affiliations:** International Laboratory of Bioinformatics, Institute of Artificial Intelligence and Digital Sciences, Faculty of Computer Science, National Research University Higher School of Economics, Moscow 101000, Russia; Bioinformatics Group, AIRI, Moscow 121170, Russia; International Laboratory of Bioinformatics, Institute of Artificial Intelligence and Digital Sciences, Faculty of Computer Science, National Research University Higher School of Economics, Moscow 101000, Russia; InsideOutBio, Charlestown, MA 02129 United States; International Laboratory of Bioinformatics, Institute of Artificial Intelligence and Digital Sciences, Faculty of Computer Science, National Research University Higher School of Economics, Moscow 101000, Russia

## Abstract

Alternative DNA conformation formed by sequences called flipons are thought to play an important role in regulating various genomic processes, either repressing or enhancing transcription, chromatin organization, DNA repair, telomere maintenance, RNA splicing, translation, and stress responses. The formation of G-quadruplexes (GQs) has been investigated experimentally using various methodologies with varying degrees of overlap between the results underscoring the need for a gold-standard GQ dataset. With this aim we trained a large language model, GQ-DNABERT using EndoQuad, the most comprehensive human GQ dataset. GQ-DNABERT recalled the training data and predicted *de novo* GQs in intergenic and intronic regions, enriched for *cis*-regulatory elements (cCREs) and ATAC-seq peaks. We evaluated the predicted GQ-DNABERT proximal enhancer–promoter (pEP) pairs, using annotations from ENdb, ENCODE, Zoonomia, Chromium multiomics scATAC-seq and scRNA-seq data from normal cells, and cCREs from normal-cancer pairs. We found GQ pEP pairs correlating with gene expression, with some pairings potentially acting as tissue-specific switches. Genes with GQ pEP pairs in cancer cells are enriched in different processes compared to the corresponding normal tissues. Overall, GQ-DNABERT is a valuable tool for extending and harmonizing data collected *ex vivo*. We demonstrate the usefulness of GQ-DNABERT for investigating transcriptional regulation in single-cell experiments.

## Graphical Abstract

**Graphical Abstract:**
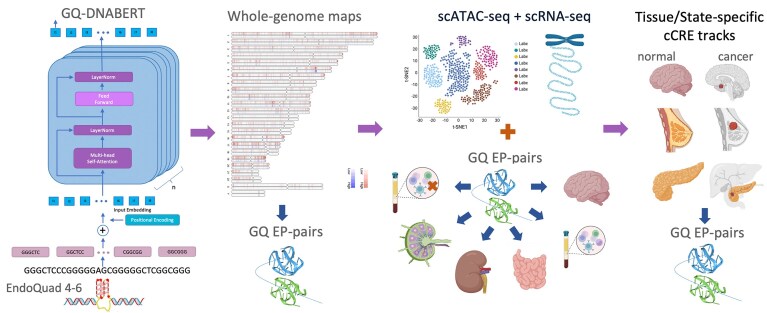

## Introduction

### GQ flipons

G-quadruplexes (GQ) are classically formed by sequences with a (G_3-5_N_1-7_)_4_ motif_._ The four-stranded structures result from the stacking of tetrads formed by the base-pairing of four guanosines around a central core that usually contains a monovalent metal ion. The four DNA strands can adopt parallel or anti-parallel orientations. Based on an analysis of almost 400 PDB structures with distinct sequences, over 49 unique GQ folds have been identified, each using a different configuration of the propeller, bulge, diagonal, and lateral loops to connect the stacked tetrads [1]. Formation of GQ by a variety of sequence motifs has been further validated by a range of experimental approaches. Some of these methods can detect specific types of GQ. For example, the BG4 antibody construct that binds only to parallel-stranded G4 structures [2]. Collectively, we refer to those sequences that can fold into both B-DNA and GQ under physiological conditions as G-flipons.

The G-flipons mapped in these prior studies reveal an enrichment in gene promoters. These sequences overlap the binding sites for many different transcription factors (TFs). In the chromatin immunoprecipitation (ChIP-seq) studies performed by the ENCODE Consortium [3], the TF-binding sites were mapped at a resolution of 150–200 base pairs. The role of GQ in transcription has been confirmed by an analysis of the P1 promoter of the MYC gene, which contains a run of five guanosine repeats and whose protein product drives many cancers. Indeed, GQ formation by the MYC promoter increases the transcription rate. Further, the most stable GQ MYC promoter fold, Pu27, promotes the highest transcription rates [4, 5]. The G-flipons in c-MYC can be replaced by GQ-forming sequences derived from other genes and placed on either DNA strand.

Overall, GQs in cells are formed transiently, with helicases capable of dismantling them. Many factors initiate GQ formation [6]. Experimental proofs of the reversibility of GQ formation include the use of fluorescently labeled reagents that have high specificity for GQs [7]. These studies reveal that GQs form during cellular stress and are rapidly resolved once cells are returned to normal conditions [8]. The flip back to B-DNA occurs in minutes or less.

### Genome-wide experimental detection methods

There have been many techniques developed for the genome-wide detection of GQ structures. One is G4-seq, which detects DNA polymerase stalling at quadruplexes formed during sequencing [9]. G4-seq detects >700 000 potential GQs. The set contains many noncanonical GQs with longer loops, and those composed of two tetrads and others with bulges formed by unpaired residues. ChIP-seq methods can be divided into approaches using antibodies to proteins that show high affinity for GQs and antibodies that bind directly to quadruplexes. G4 ChiP-seq (antibodies to GQ) identified ∼10 000 GQs in nucleosome-depleted regions but is limited by the preference of the BG4 antibody for parallel-stranded quadruplexes [2, 10]. GQs detected by this approach are enriched in the promoters and 5′ UTRs of highly transcribed genes, particularly in genes related to cancer and to somatic copy number amplifications. The CUT&Tag (cleavage under targets and tagmentation) method is based on permeabilized nuclei and extended incubation periods with the BG4 antibody-tethered Tn5 tagmentation reagent. G4 CUT&Tag method detects ∼18 000 GQs. In a side-by-side comparison, the signal-to-noise ratio was higher with this method compared to ChIP-seq approaches based on BG4 and an artificially constructed GQ-binding protein [11].

In contrast to the above approaches, chemical footprinting of GQ is performed on intact cells and is a rapid method. For example, a 70-s exposure to the reagent Permanganate/S1 nuclease footprinting of living cells is sufficient for G4 detection [12]. The chemical modifies single-stranded regions harboring different non-B DNA structures, including GQs. A motif search is then used to deconvolute the data. The technique requires GQs that incorporate thymines reactive with permanganate. Based on this approach (referred to here as KEx), ∼53 000 GQ regions are present in a human B-cell lymphoma cell line with the canonical (G_3-5_N_1-7_)_4_ motif [12].

Similar to Permanganate/S1 nuclease footprinting, N3-kethoxal–assisted labeling, called KAS-seq, is based on single-stranded DNA (ssDNA) profiles [13]. The chemical modifies guanine bases in single-stranded regions and requires exposure of cells to this reagent for 5–10 min. This method identified ∼36 000 GQs in various human cell lines. Overall, chemical mapping minimizes artefacts due to the formation of GQ following cell permeabilization and from the induction of GQ by high-affinity GQ detection reagents.

Chemical footprinting approaches require careful calibration. The first mapping studies, based on prolonged exposure to dimethyl sulfate in cells, failed to detect GQs. This reagent modifies the ssDNA produced by helicases that unwind quadruplexes, preventing GQ reformation. Over time, all G-flipons are captured in the B-DNA conformation [14]. In contrast, prolonged exposure to chemicals that stabilize quadruplexes can promote the formation of GQs not usually found in cells. Such treatments increase the rates of replication- and transcription-dependent DNA damage by preventing the flip back to B-DNA [15].

### Computational methods for G-flipon mapping

Prior to the experimental studies, regular classic pattern searches predicted over 370 000 GQ sequence motifs in the human genome, the number varying with the motif employed [16]. GQs detected by a regular pattern match were found enriched in telomeres, promoters, and 5′UTR. Several computational methods were then developed, including G4Hunter [17] and pqsfinder [18] to detect divergent G-flipons that differ both in loop and tetrad conformation. Once G4-seq data became available, machine-learning models were trained, like Quadron that is based on gradient boosting [19]. CNN models, such as PENGUINN [20] and G4detector [21] followed.

Another CNN model, DeepG4 [22], was trained on G4 ChIP-seq combined with G4-seq data and the genome-wide maps of open chromatin regions (DNase-seq and ATAC-seq). With this approach, the authors could detect cell-type specific active GQ regions and identify key TFs predictive of GQ region activity. The analysis predicted nearly 1 million G-flipons in the human genome.

### DATA aggregation

The EndoQuad database was built to combine results from different experimental GQ detection methods (G4-seq, G4 ChIP-seq, and G4 CUT&Tag), using a harmonized pipeline to call GQs in each dataset [23]. The mapping was based on pqsfinder, with ≥1 bp overlap with GQ peaks required for inclusion in the database. The loci annotated were referred to as eG4 (endogenous G4). A level of confidence was assigned to each eG4, ranging from 1 to 6. The score was on the number of datasets that provided evidence for a putative GQ fold, without conditioning on the detection method. Of 391 503 eG4, 123 150 (31.5%) were found in only one tissue. The highest level was set at 6 and corresponds to 27 376 (7%) eGQs detected in >10 samples [23]. Many of the results incorporated were from cell lines rather than tissues. The results from chemical footprinting methods were not incorporated into EndoQuad.

### Large language model in genomics

Large language models are the next generation of predictive models. In many areas, they have outperformed CNNs due to their ability to learn context. We previously applied DNABERT to identify Z-flipons in human and mouse genomes [24]. Here we apply DNABERT to predict G-flipons. As the training set, we use the data from EndoQuad database, the most comprehensive GQ database that has aggregated >1000 ChIP-seq and CUT&Tag whole-genome experiments. By training a model with this highly validated GQ dataset, we can extend GQ predictions at the genome-wide level. With this approach, we incorporate features of neighboring sequences that are conducive to GQ formation. Here, we focus on the interactions between GQ formed in promoters and proximal enhancers. We analyze published data from bulk cell and single-cell experiments, with a focus on the interactions between proximal enhancers and promoters.

## Materials and methods

### GQ-DNABERT model

To create GQ-DNABERT, we utilized a DNABERT model that had been pre-trained for 6-mer sequences (as detailed in [25]). This model was specifically adapted, or “fine-tuned,” using EndoQuad database, confidence level 4–6 [23]. The fine-tuning process extended over 10 epochs, employing the ADAM optimizer. We set the maximum learning rate at 1 × 10^−5^ and chose a batch size of 24. During the initial three epochs, the learning rate incrementally increased from zero to its maximum value. For the subsequent seven epochs, it gradually decreased back to zero.

Positives are derived directly from EndoQuad by marking bases that intersect levels 4–6 intervals on per-chromosome boolean tracks. We then tile each chromosome into nonoverlapping 512-bp windows and label a window positive if any base overlaps the positive track, negative otherwise. This produces 60 498 positive windows and 5 985 736 negative windows genome-wide. For model fitting and testing we use all positives and a 2× random sample of negatives drawn from the same genome tiling (no synthetic sequences), yielding 181 494 windows. Because windows do not overlap, no sequence is duplicated across folds and no EndoQuad interval is present in multiple folds; adjacent windows can be correlated by proximity but are assigned to exactly one fold each.

Similar to our earlier model, Z-DNABERT [24], we developed five different models. Each was trained on 80% of the available positive class examples, along with a random selection of negative class examples. We made predictions for each 512 bp region of the entire genome by averaging the outputs of models that had not encountered the data in their training phase. Thus, the final predictions represented an average from an ensemble of five models for new data and four models for previously used training data, including all experimentally identified positive regions.

Our experiments indicated that the accuracy of our predictions heavily relied on the context of the data. Consequently, for predictions encompassing the whole genome, we only used the central 128 base pairs from each 512 bp region. These pairs are surrounded by a minimum of 192 base pairs on either side, providing ample context. We achieved complete genome coverage by systematically shifting the starting point of each region by 192 base pairs. Only sequences with length ≥15 were used in further analysis.

### GQ experimental data

GQ experimental datasets used in this study were taken from the original publications: KEx [12], G4-seq [9], G4-ChIP [26], G4 CUT&Tag [11], KAS-seq [27], and EndoQuad [23].

### Building proximal EP pairs

Proximal EP pairs were built based on proximal enhancer-like sequence (pELS) annotations taken from ENCOCE cCREs and promoters based from transcripts from GENCODE annotation. ENCOCE cCREs track was downloaded from UCSC genome browser on November 2024 [28]. Zoonomia conserved *cis*-regulatory elements (cCREs) were taken from Zoonomia project [29]. Tissue/state-specific cCREs were downloaded from https://screen.wenglab.org [30]. ENdb enhancers were downloaded from the EnDB v1 (www.licpathway.net/ENdb) [31].

For transcript and gene annotation, we took GENCODE v 32 annotation as it was used in 10× Genomics multiome ATAC-seq and RNA-seq datasets to have a consistent set of promoters across all datasets analyzed. Promoter regions were defined as 1000 bp upstream and 200 bp downstream from the transcription start site (TSS).

pEP pairs were calculated as all possible combinations of transcripts with corresponding pELS as 1 bp overlap with promoter region.

### Multiome scATAC-seq + scRNA-seq analysis

Six public processed sCell Multiome ATAC + Gene Expression datasets were downloaded from 10× genomics (https://support.10xgenomics.com/single-cell-multiome-atac-gex/datasets). The samples include brain, jejunum, kidney, lymph node, PBMC, and PBMC (granulocytes removed). Dataset descriptions with corresponding links are provided in Supplementary Table S1.

10× Genomics multiome datasets are provided with the feature linkages that are defined as pairs of genomic features, such as peaks and genes, that have significant correlation in signals across cells (see description in https://www.10xgenomics.com/support/software/cell-ranger-arc/latest/algorithms-overview/algorithms-for-computation-of-feature-linkages). For ATAC-seq + RNA-seq data, we took the preprocessed feature linkage data that provided correlation between ATAC-seq peaks and gene expression. For analysis of GQ pEP pairs correlation with gene expression, we took ATAC peaks with the absolute value of correlation over threshold and overlapped them with pELS and promoters (regions 1000 upstream and 200 downstream from TSS). The results in the main text are presented with the correlation over 0.2; the results of the analysis with ATAC correlation over 0.5 are presented in Supplementary Table S2.

### Cancer and normal tissue

Tissue and state-specific cCRE tracks for normal-cancer pairs from brain, breast, and pancreas were taken from ENCODE (see the name and accession numbers for each sample in Supplementary Table S3).

### GO-enrichment analysis

GO-enrichment analysis was done with the Database for Annotation, Visualization, and Integrated Discovery (DAVID) tool [32].

### Statistical analysis

Association of promters, proximal enhancers, and pEP pairs with GQs and open chromatin (ATAC-seq peaks) was assessed with Fisher-test (see calculations in Supplementary Table S1),

## Results

### GQ-DNABERT model

**Figure 1.**
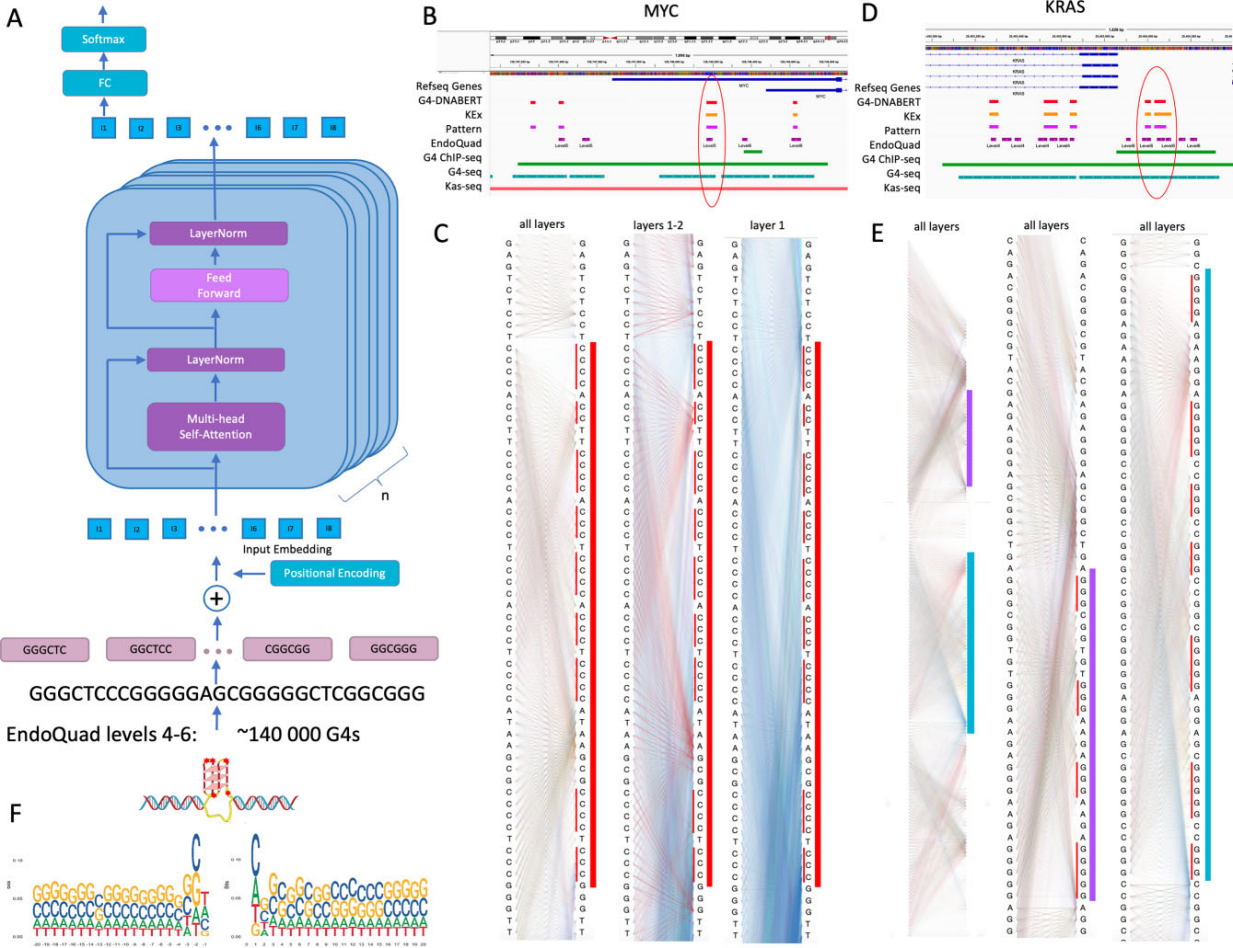
GQ-DNABERT model. (**A**) Architecture of GQ-DNABERT model. It was made by fine-tuning DNABERT using EndoQuad levels 4–6 data. (**B**) Quadruplexes at MYC promoters predicted by different methods. (**C**) Quadruplexes at KRAS promoters predicted by different methods. (**D**) Attention maps of the region around the famous MYC GQ P1 PU27 promoter encircled in red in Fig. 1B. Left columns is the summary view from all 12 attention layers; middle column is the combination of layer 1 and 2; right column is an attention map from layer 1. The bar to the right indicates GQ. (**E**) Attention maps of the region with two GQs at KRAS promoter (encircled in Fig. 2D). Th left column depicts the summary view of all 12 attention layers; middle column is the attention map from all layers around the first GQ; right column is the attention map from all layers around the second G4. Bars to the right indicate GQs. (**F**) Logo of 20 bp flanks around GQs predicted by GQ-DNABERT.

GQ-DNABERT was created by fine-tuning the DNABERT [25] model that is trained on a 6-mers representation of DNA sequence within a 512 bp context (Fig. 1A and see details in the “Materials and methods” section). We used the EndoQuad database, the most comprehensive GQ dataset comprising almost 400 000 GQs from >1000 ChIP-seq and CUT&Tag whole-genome experiments. Each GQ from EndoQuad database is assigned to a level of confidence based on the number of experiments that have confirmed a particular flipon can adopt the GQ conformation. For training, we chose levels 4–6 (∼140 000 GQs), which means that GQ formation was confirmed by at least 4 or more experiments. GQ-DNABERT achieved recall of 0.9993 and precision = 0.9977 on the test set (Supplementary Table S4). We then used the trained GQ-DNABERT model to perform whole-genome inference (see “Materials and methods” section) and predicted ∼360 000 G-flipons in the human genome. When scanning the entire genome with the minimum length of 6 and a decision threshold of 0.25, we obtain precision = 0.315, recall = 0.9866, F1 = 0.477, accuracy = 0.998, and ROC–AUC = 0.992 (Supplementary Table S4). These values demonstrate that, while ranking is strong (high ROC–AUC, high recall), the task is not made trivial by our negative design. Because negatives are real genomic windows sampled from the same chromosomes and tiling procedure, they naturally reflect genomic GC and repeat structure and include GQ-like patterns that are not annotated by EndoQuad.

The main advantage of a transformer-based model is that it can learn the context of a genomic element, as the attention scores generated reveal the sequences used by the model to predict the regions of interest. High scores provide contextual information, as illustrated by the attention maps generated for the G-flipons present in the MYC (Fig. 1B and C) and KRAS (Fig. 1D and E) promoters. The attention maps for the famous MYC GQ P1 PU27 promoter [5, 33, 34] (Fig. 1C) have high scores not only for GQ regions but also for the adjacent sequences. Attention maps around the two GQs in the KRAS promoter [35, 36] (Fig. 1D) also reveal that high attention scores are found at the boundaries of each GQ region (Fig. 1E). The ability of language models to learn the context of functional regions thus produces a more informed selection of what otherwise is a seemingly simple GQ motif search. A genome-wide ranking for all 6-mers according to their GQ-DNABERT attention scores and their frequency in the human genome is given in Supplementary Table S5.

### GQ-DNABERT model revealed novel GQs in noncoding regulatory regions

**Figure 2.**
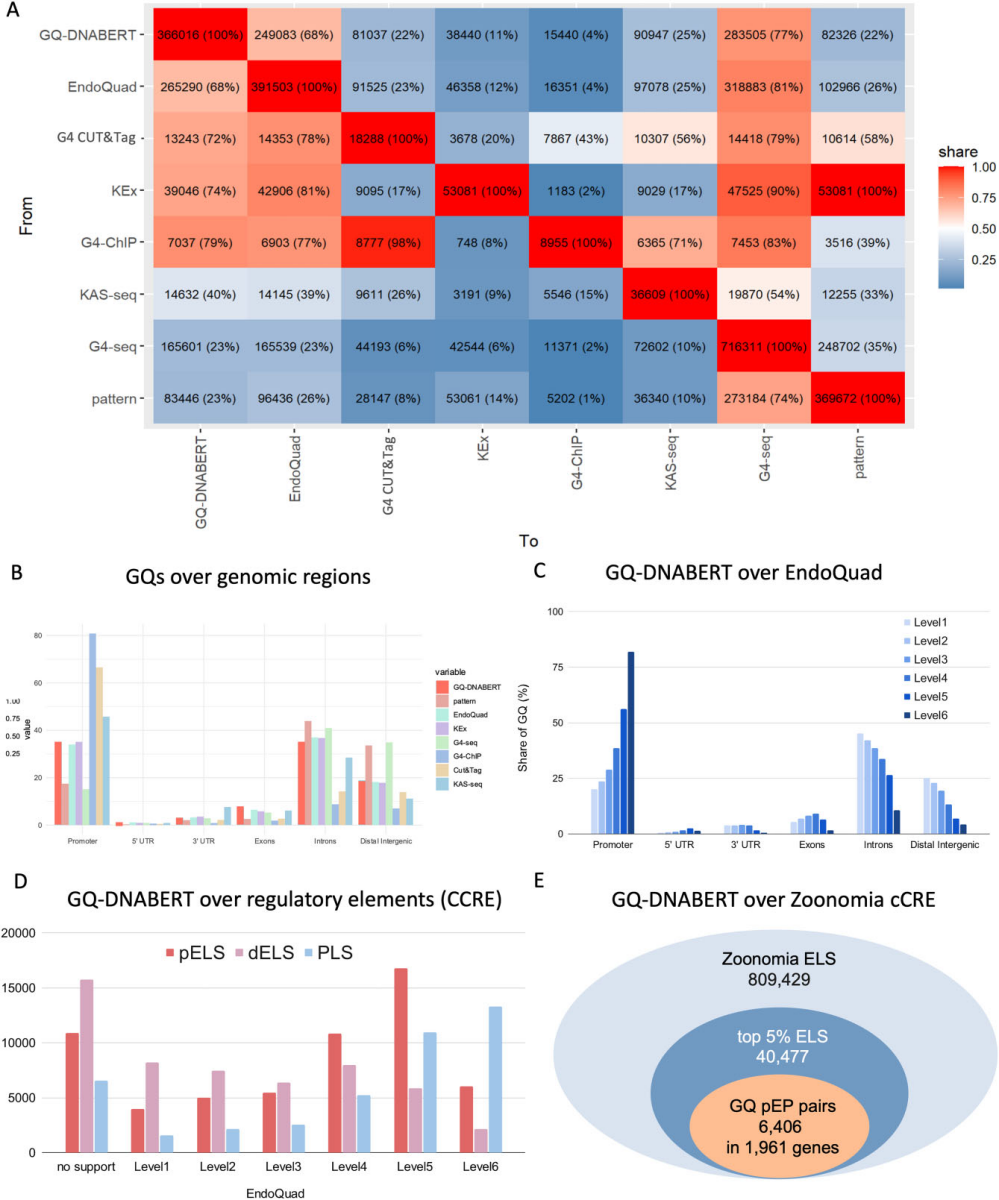
GQ-DNABERT comparison with other methods. (**A**) Genome-wide comparison of GQ-DNABERT and other experimental GQ-detection methods. (**B**) Comparison of GQ-DNABERT with other experimental GQ-detection methods over genomic regions. (**C**) Overlap of GQ-DNABERT with EndoQuad database stratified by the confidence levels. (**D**) Overlap of GQ-DNABERT with EndoQuad and ENCODE candidate cCREs. (**E**) GQ pEP pairs in conserved cCRE from Zoonomia project.

It has been known that different GQ detection methods such as G4-seq, G4 ChIP-seq, G4 CUT&Tag, KEx, and KAS-seq show little overlap when considered at the whole-genome level. Comparison of GQ-DNABERT with all experimental techniques, EndoQuad database and computational pattern search is given in Fig. 2A. The results from pairwise overlaps of eight different methods confirm the poor and varying overlap from 6% to 97% of the current experimental datasets.

The distribution of GQs over genomic regions, as predicted by the different methods, is given in Fig. 2B. As expected, G4 ChIP-seq and G4 CUT&Tag are enriched in promoters, with almost 80% and 65% of predictions falling into these regions. GQ-DNABERT, KEx, G4-seq, and EndoQuad have almost an equal share of GQ in introns—around 40%. G4-seq has the highest share in intergenic regions (37%), where enhancer sequences are typically present. Classical pattern matching and GQ-DNABERT detect fewer GQs in promoters than G4-seq. At the same time, G4 ChIP-seq and G4 CUT&Tag find a lower frequency of GQs in introns and intergenic regions than found with other methods.

The distribution of EndoQuad and GQ-DNABERT across different genomic regions is similar. In total, only 68% of GQ-DNABERT predictions overlap those in the entire EndoQuad set (Fig. 2A), although for levels 5 and 6 the overlap was 93% and 95%, respectively (Supplementary Table S6). The GQ-DNABERT results suggest that not all functional GQs in the genome have been experimentally mapped. One likely reason is the absence of experimental data for some tissues that form GQ in a tissue-specific manner. Indeed, almost half (47%) of the experiments in EndoQuad are derived from human embryonic kidney cells (HEK293 T). Many other datasets are from A549, K562, and H1975 cells (Supplementary Fig. S1). Another possibility is that some GQs cycle more rapidly than others, making them more challenging to identify in the mixed cell populations typically assayed in ChIP-seq experiments.

A comparison of the GQ-DNABERT overlap with the different EndoQuad confidence levels, stratified by genomic region, is given in Fig. 2C. The overlap for promoter GQs between GQ-DNABERT predictions increases with the number of experimental replications recorded in EndoQuad. The maximum concordance is ∼80% for Level 6 GQs. The inverse trend is observed for intronic and intergenic GQs, where most GQs have only level 1 support in EndoQuad. Many of the GQ-DNABERT predictions without EndoQuad support are located in noncoding regions, again reflecting the sparsity of datasets that cover these sequences. Consequently, many GQ-DNABERT predictions require further experimental validation. However, the overlap of other predictions with well-verified EndoQuad annotations increases the *a priori* probability that most of the GQs predicted by GQ-DNABERT are formed in cells.

### GQ-DNABERT GQs in promoter–enhancer pairs

ENCODE candidate *cis-*regulatory elements

To further explore the regulatory potential of GQs, we overlapped GQ-DNABERT predictions with cCRE from the ENCODE project [28]. A total of 59 053 (16.15%) GQ-DNABERT predictions intersect with proximal enhancers (pELSs; 6-fold enrichment, *P*< 0.001, permutation test), and almost an equal number, 53 750 (15.7%) of GQs (2-fold enrichment, *P*< 0.001, permutation test) map to distal enhancers [distal enhancer-like sequences (dELSs)]. The overlap of GQ-DNABERT with PLS, pELS and dELS, and EndoQuad is given in Fig. 2D and Supplementary Table S7. Here, our analysis focuses solely on the modulation of gene transcription by GQs formed in pELS and their promoters. Other interactions that involve dELS will be examined in a subsequent paper.

### ENdb

We first examined the ENdb, a manually curated enhancer database [31], in which promoter–enhancer interactions in cell lines are experimentally verified. The database contains data for 425 enhancers that are connected to 159 genes. We were interested in genes and transcripts in the collection that have experimentally validated G-flipons in the region ± 100 bp surrounding both the enhancers and the promoter pairs. The size of this 200 bp segment corresponds to that of a DNase hypersensitivity site, as defined by the ENCODE consortium [37]. By focusing on proximal enhancers located within 1 kb of a TSS, we identified 37 pELS that map to 35 genes. Based on all available transcripts for each gene, we obtained 163 proximal enhancer–promoter (pEP) pairs, as each gene can have multiple pEP pairs. We then looked for the overlap of these pEP pairs with GQ predicted by GQ-DNABERT. We identified 195 GQs (89 in promoters and 106 in enhancers) that corresponded to 80 GQ pEP pairs or ∼ 50% of the annotated pEP pairs (Supplementary Table S8). Of those genes with a GQ directing overlapping a pELS sequence, 14 of 16 GQ formed an pEP pair with the nearest gene. Of this small subset, 10 genes are involved in cell death regulation according to DAVID (The false discovery rate (FDR) = 8.6 × 10^−5^). Such proteins are capable of rapidly terminating cells in which G-flipons are frozen in the GQ conformation, either due to DNA damage or defective helicase function (Supplementary Table S8).

### Zoonomia conserved cCREs

To further understand GQ pEP pairs, we examined data from the Zoonomia project that examines over one million cCREs across 241 mammals [29]. The total number of enhancer-like sequences (ELS) regions in this database is 809 429, from which we took the top 5% human ELS (by phylop score, 40 477) and checked for the overlap with GQ-DNABERT predictions. We then identified those enhancers that fell within the proximal promoter region and searched for the subset associated with GQ predictions in the PLS (Supplementary Table S7). For the 7091 top 5% conserved pELS, we identified 6406 GQ pEP pairs corresponding to 1961 genes (Fig. 2E and Table 1). EndoQuad supports the GQ pEP pairs in 1923 of these genes (97%). Of the rest, only 38 genes with GQ pEP pairs are uniquely predicted by GQ-DNABERT. Enrichment analysis of the 1961 genes revealed the following GO-categories: GO:0048568 embryonic organ development (FDR = 1.26 × 10^−09^), GO:0007389 pattern specification process (FDR = 2.76 × 10^−09^), GO:0048705 skeletal system morphogenesis (FDR = 7.63 × 10^−08^), GO:0048706 embryonic skeletal system development (FDR = 2.02 × 10^−06^), GO:0045165 cell fate commitment (FDR = 2.99 × 10^−06^), and GO:0060537 muscle tissue development (FDR = 6.11 × 10^−06^). The full list of GO-enrichment categories is given in Supplementary Table S7. The results are consistent with an essential role for GQ pEP pairs in gene regulation and, more specifically, in embryonic development and tissue specification.

**Table 1.** GQ enhancer–promoter pairs detected with different methods

Region	Set	GQ-DNABERT in ENCODE cCRE	GQ-DNABERT in Zoonomia cCRE	EndoQuad In ENCODE cCRE	EndoQuad in Zoonomia cCRE
GQ present in the promoter of a pEP pair by the total number of pairs and by the total number of genes	Pairs	169 020/212 825 (79.42%)	9633/11 662 (82.6%)	167 840/212 825 (78.86%)	9585/11 662 (82.19%)
	Genes	17 739/24 142 (73.48%)	2874/3441 (83.52%)	17 565/24 142 (72.76%)	2856/3441 (83%)
GQ present in the enhancer of a pEP pair by the total number of pairs and by the total number of genes	Pairs	75 254/212 825 (35.36%)	6724/11 662 (57.66%)	78 553/212 825 (36.91%)	6774/11 662 (58.09%)
	Genes	13 045/24 142 (54.03%)	2057/3441 (59.78%)	13 184/24 142 (54.61%)	2049/3 441 (59.55%)
GQ present in both the enhancer and promoter of a pEP pair by the total number of pairs and by the total number of genes	Pairs	71 799/212 825 (33.74%)	6406/11 662 (54.93%)	74 703/212 825 (35.1%)	6458/11 662 (55.38%)
	Genes	12 318/24 142 (51.02%)	1961/3 441 (56.99%)	12 410/24 142 (51.4%)	1961/3 441 (56.99%)
GQ count in promoters of GQ pEP pairs/all pEP pairs	GQ	73 622/88 926 (82.79%)	11 711/14 953 (78.32%)	82 231/97 258 (84.55%)	13 082/16 359 (79.97%)
GQ count in enhancers of GQ pEP pairs/all pEP pairs	GQ	36 683/37 657 (97.41%)	3508/3605 (97.31%)	40 134/41 173 (97.48%)	3775/3860 (97.8%)
GQ count in pEP pairs / all pEP pairs	GQ	78 112/93 659 (83.4%)	12 216/15 484 (78.89%)	87 319/102 617 (85.09%)	13 631/16 939 (80.47%)

### GQ pEP pairs in multiome scATAC-seq + scRNA-seq

**Figure 3.**
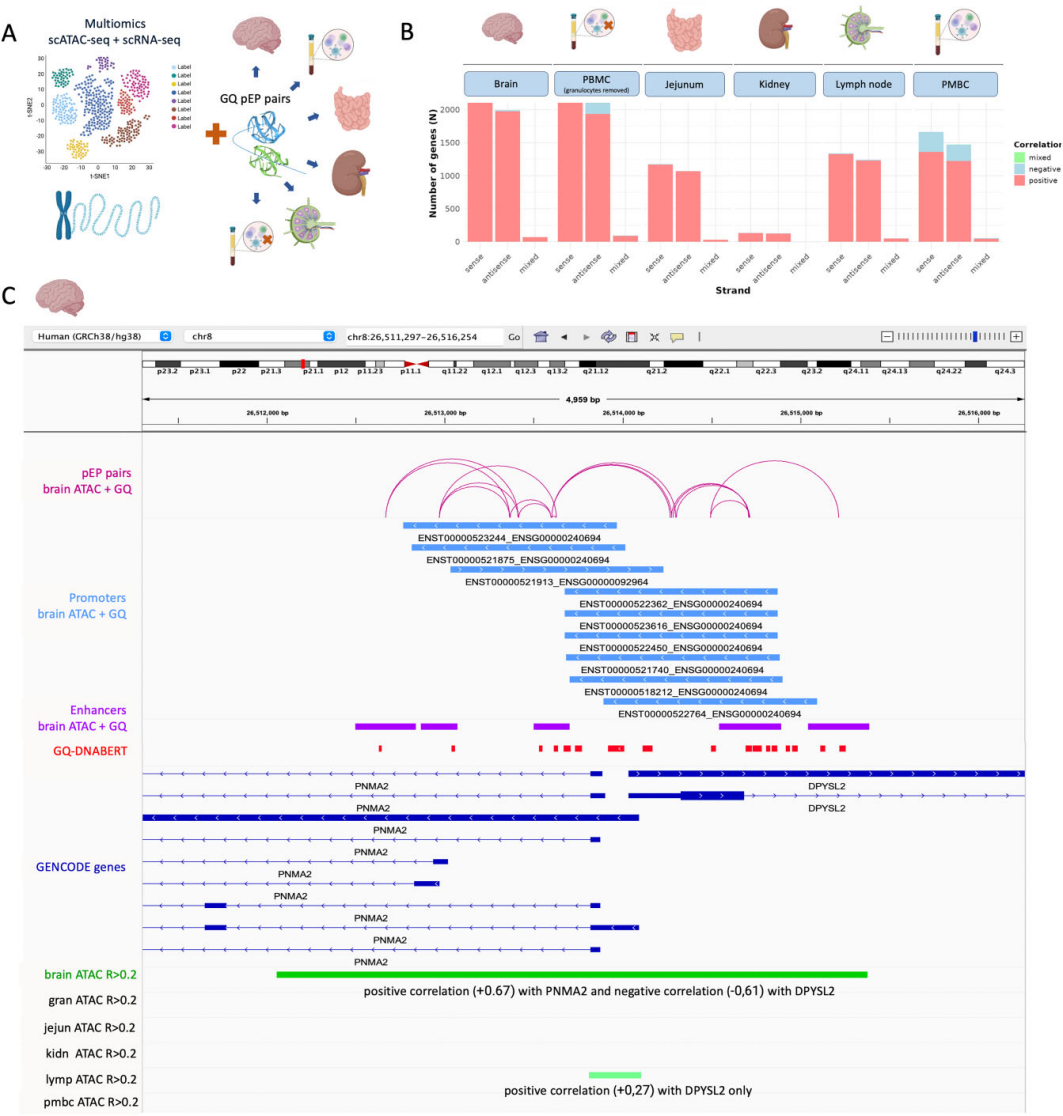
GQ pEP pairs in multiome scATAC-seq + scRNA-seq. (**A**) General schema of GQ pEP pair analysis. (**B**) pEP pairs are present in positively correlated ATAC peaks independently of strand. (**C**) GQ pEP pairs in DPYSL2–PNMA2 promoter region that have discordant effect on expression of DPYSL2 and PNMA2 genes in brain multiomics scATAC-seq + RNA-seq data. The figure uses elements created in BioRender.com.

Single-cell GQ detection methods provide information about G-flipon function that is lost in the bulk sequencing of cell populations. To examine GQs present in the nucleosome-depleted, active regions of the genome, we explored single-cell data generated using the Chromium assay kit developed by 10× Genomics. This multiome method yields ATAC-seq and quantitative gene expression data from the same cell (Fig. 3A). We examined datasets from six different tissues (Supplementary Table S1). Four sets were derived from normal tissue [brain, peripheral blood monocytic cells (PBMCs), PBMC without granulocytes, and jejunum] and two from tumors (kidney and lymph node). We assessed the overlap between GQ-DNABERT-predicted GQs in promoters and pELS, as defined with ENCODE cCRE marks. We also queried whether these G-flipons were present in the open chromatin identified by ATAC-seq to assess the functionality of the predicted GQs.

We found that in the brain sample, promoters for 2838 (27%) of the annotated genes were located in open chromatin regions, and among them, 1386 (49%) promoters had GQs (Supplementary Table S1). For PBMC, ∼43% of promoters were located in open chromatin. Approximately 24% of these promoters also had GQ-DNABERT predictions. On average, 30% of promoters from all six tissue samples had open chromatin regions that overlap GQ-DNABERT predictions (see Supplementary Table S1). The number of pELS in open chromatin varied from 1% in the renal cancer sample to 43% in PBMC (with granulocytes removed). Of these pELS, 54% in renal cancer and 51% in PBMC (with granulocytes removed) overlapped predicted GQs. The corresponding number of GQ pEP pairs in open chromatin regions in the different samples thus varies accordingly: 18 861 for PBMC (with granulocytes removed), 824 for the renal cancer sample, 11 200 for the lymph node cancer sample, 15 434 for brain, 7374 for breast, and 10 893 for PBMC (see the details in Supplementary Table S1).

We also calculated the number of expected GQ pEP pairs by multiplying the frequency of predicted GQs in enhancer and promoter regions. We found a slight excess of GQ pEP pairs in the observed data, with enrichment varies from minimum 1.14 (PBMC, granulocytes removed) to maximum 1.69 (kidney, cancer sample) (see Supplementary Table S1 for other tissues), providing consistent but not conclusive evidence for the interaction between GQ promoter and proximal enhancer pairs. The result represents only a snapshot of each cell at the time of data collection. One interpretation is that at any one time, only a small portion of the population is actively forming pEP pairs. If these pairs are functional, then the pEP pairs should correlate with a particular outcome.

To examine the role of pEP pairs in transcription, we leveraged the RNA-seq results collected from the same cells using the Chromium assay kit (Supplementary Table S2). The positive or negative correlation between ATAC-seq peaks and RNA expression of a nearby gene located on the same chromosome is provided as metadata within each sample. We asked whether the formation of GQ pEP pairs increased or decreased gene expression. We found that GQ pEP pairs were positively correlated with transcription in 90% of cases. This finding was true regardless of whether the pEP pair was on the coding or noncoding strand relative to the direction of transcription (Fig. 3B and Supplementary Table S2). Notably, the brain and PBMC pEP pairs are enriched in tissue-specific processes (Supplementary Table S1).

Given the potential of enhancers to pair with more than one promoter and thereby impact the expression of adjacent genes, we looked for cases where a GQ pELS in open chromatin correlated positively with the expression of one gene, and negatively with that of an adjacent gene, i.e. we tested whether a pELS lying between the PLS of two different genes had a discordant effect on their transcription. In particular, we were curious as to whether such enhancers could be involved in switching on the expression of one gene during differentiation, while turning off the transcription of an adjacent gene. To assess this possibility, we asked whether this class of enhancers controls gene expression in a tissue-specific manner. As shown in the Venn diagram for six tissues in Supplementary Fig. S2, the answer was yes. A small class of tissue-specific enhancers forms GQ, which either increases or decreases gene expression, while having the opposite effect on transcription from an adjacent promoter. This outcome is observed even when both PLS incorporate flipons capable of forming GQs.

An example of GQ pEP pairs that positively regulate one gene while suppressing the transcription of another involves the DPYSL2–PNMA2 promoter region (Fig. 3C). This region has promoters and enhancers with GQs that can form GQ pEP pairs, as depicted by the arcs in the upper track. However, only the brain ATAC-seq region has a positive (+0.67) correlation with PNMA2 (a capsid-forming protein associated with paraneoplastic neurological disorders) and a negative correlation (−0.61) with DPYSL2 (plays a role in neuronal development and polarity, as well as in axon growth and guidance). In contrast, ATAC-seq results for the same region in lymph node cancer correlated only with the expression of DPYSL2, not with PNMA2. The correlation coefficient was much lower and in the opposite direction (+0.27). No ATAC-seq peaks were observed in this region in other tissues.

### GQ pEP pairs in cancer versus normal tissue

**Figure 4.**
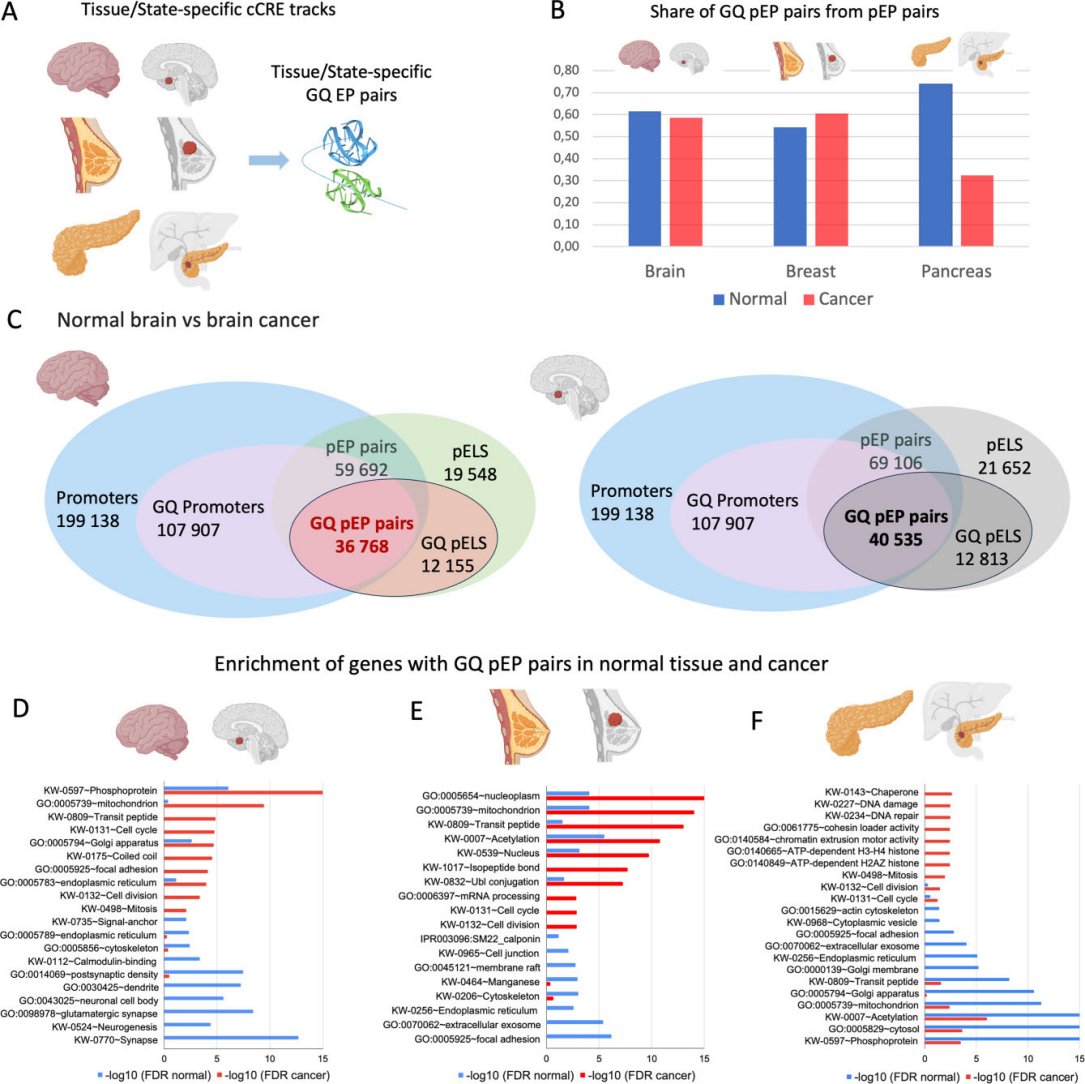
GQ pEP pairs in cancer tissue. (**A**) General schema of the analysis that includes tissue and state-specific ENCODE cCRE tracks for normal-cancer pairs. (**B**) Share of pEP pairs with GQ in both PLS and pELS relative to the total number of pEP pairs. (**C**) Venn diagram for the number of promoters, proximal enhancers, and pEP pairs with GQ in cancer and matched normal tissues. (**D–F**) GO-Enrichment analysis of genes with GQ pEP pairs in normal and cancer samples for (D) brain, (E) breast, and (F) pancreas. All categories were checked for both samples: if one gene set was enriched in one sample and not significantly enriched in the other sample, then the enrichment was depicted only for one. The figure uses elements created in BioRender.com.

We also examined how the GQ pEP pair landscape changes in cancer compared to normal tissue. We made the comparison using a cell type in normal tissue matched to that of the tumor. For this analysis, we utilized tissue- and state-specific ENCODE cCRE tracks for normal-cancer pairs from the brain, breast, and pancreas, and calculated the number of potential GQ pEP pairs (Fig. 4A and B, and Supplementary Table S3). For brain and breast, we observed approximately an equal number of GQ pEP pairs in normal versus cancer tissue states (Fig. 4B). In contrast, pancreatic cancer cells showed a significantly lower share of GQ pEP pairs compared to normal tissues, suggesting that the pairing of PLS with pELS is disrupted in this tumor type.

GO analysis reveals significant differences in the processes and functions of genes that form GQ pEP pairs in normal compared to cancer cells (Fig. 4D–F and Supplementary Table S3). Thus, genes with GQ pEP pairs in brain, breast, and pancreas cancers are enriched for mitochondrion, cell division, cell cycle, mitosis, and transit peptide categories (Fig. 4D–F with the complete list of enriched categories for each tissue given in Supplementary Table S3). In contrast, for matched normal tissue cells, we observe enrichment in tissue-specific processes. Thus, for brain, these are the categories: synapse, neurogenesis, calmodulin binding, dendrites, and others (Fig. 4D and Supplementary Table S3); for breast, focal adhesion, extracellular exosome, manganese, calponin, and others (Fig. 4E and Supplementary Table S3); for pancreas, acetylation, phosphoproteins, and Golgi apparatus (Fig. 4F and Supplementary Table S3). In all three normal tissues, cytoskeleton and endoplasmic reticulum genes with GQ pEP pairs appeared to be significantly enriched, possibly reflecting that these cells have a secretory phenotype. Overall, our results suggest that different sets of GQ pEP pairs are activated to initiate tissue-specific processes in normal tissues. In contrast, cancer cells turn on pathways that are essential for cell division and growth.

## Discussion

### Gold-standard whole-genome GQ annotation

One of the goals in GQ research is to reconstruct the most confident whole-genome map of functional GQs in the human genome. GQs are transient structures that are formed to initiate or suppress genetic programs. As part of the cycle, they are then resolved by helicases to reform double-stranded B-DNA. Any experimental technique can only capture the GQ subset present in a cell at the time the sample was collected. Some of the GQs identified may be specific for a particular cell type. The formation of other GQs may depend on the context. Such limitations are particularly true for mixed cell populations, where only a small fraction may be actively forming GQ at a particular locus. Consequently, in many experiments, it can be challenging to estimate the fraction of flipons folded as GQ given that many more are currently in the B-DNA conformation.

Further, each experimental method for detection of GQs has unique limitations that may contribute to the disparate results obtained from analysis of a particular cell type (see [38] for a review of the strengths and weaknesses of different GQ detection methods). These problems are highlighted by the data presented in Fig. 2A. The G4-seq method is based on *in vitro* sequencing of DNA after it is extracted from cells. The technique relies on the difference in DNA conformation resulting from the use of K^+^ or Li^+^ in sequencing buffers. In this situation, K^+^ but not Li^+^ favors GQ formation. The approach detects >700 000 GQs, of which an unknown number may represent noise in the sequence data or GQ that are no-functional inside cells. G4 ChIP-seq and CUT&Tag detect an order of magnitude less GQ (∼10 000), but both depend on an antibody that binds preferentially to a GQ parallel strand orientation [2]. KEx dataset is based on the rapid chemical modification of single-stranded DNA in intact cells. The analysis yields ∼50 000 GQ, which are computational predictions based on the presence of a GQ motif within any single-stranded regions detected. The method itself has other limitations, as it requires unpaired thymines at the site of GQ formation. ChIP-seq and CUT&Tag experiments for GQ detection are enriched in promoters. In many cases, intergenic and intronic GQs, especially those embedded within enhancer and promoter condensates, may be challenging to detect as they may not be accessible to the antibodies and other GQ-detection reagents used in these experiments.

The mapping of GQ may be biased in other ways. For example, several factors influence the detection promoter GQ. Previous studies on transcriptional complexes have revealed the rapid loss of enhancer–promoter contacts immediately after a transcriptional burst (Bartman, Hsu *et al.* 2016). The immediate disassembly of condensates following a transcriptional burst enables the rapid reset of the promoters but limits the times at which GQ can be detected [39]. The ability of promoters to interact with other genomic regions, such as splice sites and 3′ UTR elements, may instead facilitate the detection of promoter GQ [40]. Under these conditions, the involvement of promoters in various RNA transactions, such as looping, splicing, and transcript termination, may depend on GQ formation by the different genomic regions involved [41]. In contrast, the transient nature of GQ formation in nonpromoter regions may make these structures harder to map than the more promiscuous partnering of promoter GQ. Their interaction with promoter GQs may exist only long enough to specify a particular splice or to trigger use of a specific transcript termination site. In other cases, the formation of GQ by these other regions may be brief and only necessary to prevent DNA methylation of CTCF-binding sites by DNA methyltransferase 1 (encoded by DNMT1). The unmethylated sites then remain available to reform the CTCF-dependent promoter loops that were disrupted as RNA polymerases transcribed through that region [42]. The known signal-to-noise issues inherent in certain ChIP-seq methods further complicate the detection of these transiently formed GQs [41]. Overall, the available experimental data indicate that there is a higher probability of mapping a promoter GQ experimentally than GQ in other genomic regions (Fig. 2B).

As we demonstrate with GQ-DNABERT, large language models can help overcome these experimental mapping problems. They can extend existing data to predict novel sites of GQ formation in sequences that have the same signature as found in the training dataset. These predictions are heavily dependent on the quality of the training data. The validated GQ annotated at levels 4–6 of EndoQuad has played a crucial role in building the GQ-DNABERT model. As of today, the EndoQuad database has incorporated over 1000 experiments. The high-confidence GQs, defined by levels 4–6, can be considered an experimental gold standard. Nevertheless, as noted above, the data collected in EndoQuad have their limitations. Almost half (47%) of the experiments in EndoQuad are derived from human embryonic kidney cells (HEK293 T). Many other datasets are from A549, K562, and H1975 cells (Supplementary Fig. S1). Consequently, the database lacks information from a broad range of tissues. When present, the tissue-specific data are often from a single experiment. This limitation partially explains why only 68% of GQ-DNABERT predictions overlap with the full EndoQuad dataset, highlighting the need to map GQs in more tissue samples.

Besides predicting GQs, GQ-DNABERT also provides insights into the implicit patterns, both in loops and in adjacent sequences, that affect the propensity for a G-flipon to form a GQ. This information is provided by the attention maps that GQ-DNABERT generates. In particular, GQ-DNABERT pays attention to the 20 bp flanking GQ structures, and an excessive number of GGG repeats is observed in MYC (eight blocks) and KRAS (seven blocks) promoters. A different bioinformatic approach has previously identified the enrichment of such patterns in promoters [43]. The context information embedded in GQ-DNABERT helps increase the accuracy of predicting sites with biological relevance compared to methods that rely solely on identifying a motif capable of forming three or more tetrads.

Taking together that GQ-DNABERT was trained on the most comprehensive collection of more than 1000 GQ-detection experiments, where each GQ has support of more than four experiments, and that it obtained an F1 score of 0.9985 on the test set, the GQ-DNABERT generated whole-genome predictions can be considered as an *in silico* generated gold-standard map of GQs. Notably, GQ-DNABERT overlaps with G4-seq (77%), which does not depend on any tissue or cell-type-specific factors. GQ-DNABERT also overlaps with the KEx dataset (74%). The KEx data are not included in EndoQuad. In contrast to the other GQ detection methods, GQ is ascertained from living cells under physiological conditions. The approach reduces the risk of detecting GQ formed during the extraction of DNA from cells or in any of the subsequent processing steps.

### Emerging role of GQs in Gene Transcription

The major finding resulting from the analysis of GQ-DNABERT predictions is the coordinate formation of GQ in pELS and PLS pairs, as observed in single-cell experiments. A role of GQs in proximal enhancers has been shown earlier for different proteins that can bind GQs, such as SP1 [44], CNBP [45], MAZ [5, 46], and others [47, 48]. YY1 (Yin Yang 1) TF binds to GQ in pELS and forms dimers that can bridge long-range DNA interactions with more distant DNA regions [49].

The role of enhancer GQs has been shown in developping drug resistance in ovarian cancer with ChIP-seq, CUT&Tag, ATAC-seq, and RNA-seq whole-genome experiments in paired drug-sensitive and resistant cell lines [50]. It appeared that the overall number of GQs decreased almost in half in drug-resistant cells (from ∼13 000 to 6000) but looking only at promoter and enhancer regions the authors obsereved a drop in promoters and an increase at intronic and intergenic regions. Additional ATAC-seq, RNA-seq and H3K27ac confirmed GQ localization in enhancer regions and found correlation with elevated gene expression in pathways linked to resistance (WNT and Hippo signaling). Enrichment of GQs in EP pairs was also confirmed in [51] in HepG2 cells where the authors showed that over 60% of RNA polymerase II mediated DNA loops in HepG2 cells overlap with GQs, and genes with GQ in EP-pairs have leveated level of expression.

An unexplored subject is multimolecular GQs that can play an important role into liquid–liquid phase (LLP) separations [52]. Intronic GQ clusters can act as superenhancers and initiate LLPS in the absence of proteins. Additionally long-range multimolecular G4s, could play a key role in chromatin looping. Nucleolar localized Cockayne Syndrome B (CSB) protein binds to intermolecular GQs formed in ribosomal DNA (rDNA), while showing negligible binding to intramolecular GQs or single-stranded DNA [53]. This finding highlights the potential role of R-loop-associated GQs in nucleolar organization and aging-related pathologies.

Overall, the results based on GQ-DNABERT are in line with these reported findings. Many of the predicted GQs in proximal enhancers lie within open chromatin regions that are associated with gene transcription. The analysis suggests that pELS GQs pair with GQ in PLS to modulate gene expression, regardless of which strand they form on. The pEP pairs that form differ between normal and cancer cells. The analysis of multiome single-cell ATAC-seq and RNA-seq data further suggests that many pEP pairs are tissue specific.

A role of GQ in tissue-specific processes is largely unexplored experimentally, but overall is supported by our analysis here and a small number of experimental studies (Bashkatov *et al.* 2025, Herbert, Pavlov *et al.* 2023). Previously, the enrichment of GQ in neurological tissues, as detected by CUT&Tag, has been reported (Spiegel, Cuesta *et al.*). GQs have also been detected by G4 CUT&Tag on mouse embryonic stem cells and localized to active promoters (characterized by the presence of eRNAs and both H3K4me1 and H3K27ac histone marks) and poised enhancers (H3K4me1 and of H3Kme3 marks), but not at primed enhancers (with only H3K4me1 marks) [41]. Thus, the presence of GQ was proposed to be a functional structure that distinguishes active from primed enhancers. GQs were lost from embryonic primed enhancers upon differentiation to neural progenitor cells and then replaced by tissue-specific GQs. The study further revealed that R-loop CUT&Tag at TSS are highly correlated with G4 CUT&Tag, but that transcription is not required to stabilize GQ formation [41]. Instead, the authors propose that GQ is formed by an interaction between a pair of G-repeats from a promoter with a pair of G-repeats from an enhancer. We provide evidence here for the involvement of pELS and PLS promoter pairs in these processes.

## Conclusions

The GQ-DNABERT model is trained on hundreds of G4 ChIP-seq and G4 CUT&Tag datasets, using results replicated in at least four independent experiments. The model extends the trained datasets and generates a whole-genome map for humans. This mapping can be considered the most reliable currently available, given that it is trained on almost all the available and highly validated whole-genome GQ mapping experiments. We demonstrate that the GQ-DNABERT predictions are further validated by the KEx dataset, which is not included in EndoQuad but instead maps GQ in intact cells using chemical footprinting. GQ-DNABERT overcomes method-specific limitations of existing experimental datasets and generates novel predictions, which should be taken with caution until further experimental validation.

Indeed, GQ-DNABERT reveals many novel GQs in noncoding regions that are enriched in regulatory elements, with >6-fold enrichment in proximal enhancers. After training GQ-DNABERT, we evaluated the potential functional roles of predicted GQ pEP pairs using experimentally verified interactions from a diverse set of databases, including ENdb, ENCODE cCREs, Zoonomia cCREs conserved in humans and other mammals, chromium multiome ATAC-seq and RNA-seq datasets of normal and cancer cells, and three normal-cancer pairs with tissue/state-specific cCREs. By overlapping these datasets with GQ-DNABERT predictions, we identified a subset of pEP pairs that direct the tissue-specific and cancer-specific expression of genes. The use of GQ-DNABERT in this manner leads to experimentally testable hypotheses. The results obtained from such explorations will help further our understanding of the role GQs play in tissue-specific gene expression.

The GQ-DNABERT whole-genome maps for humans have applications beyond the pEP pairs described here. Elucidating the functions of intronic and intergenic GQs is an ongoing area of research. Active areas of investigation include the formation of GQ clusters in super-enhancers, LLP separation [52], chromatin remodeling, and tissue-specific programs in which small RNAs modulate GQ formation [54, 55]. The development of new therapeutics to reprogram diseased cells will be helped by improving our understanding of how flipons, such as those that form GQ, impact cell fate.

## Supplementary Material

gkaf1007_Supplemental_Files

## Data Availability

The code for GQ-DNABERT model is freely available at https://github.com/mitiau/G-DNABERT and https://zenodo.org/doi/10.5281/zenodo.13748468.
