# Supplementary material for: GQ-DNABERT reveals GQ proximal enhancer–promoter interactions associated with tissue-specific transcription
Source: Nucleic Acids Res. 2025 Oct 14;53(19):gkaf1007. doi: 10.1093/nar/gkaf1007 (PMC12526042; doi:10.1093/nar/gkaf1007)
Supplement: gkaf1007_Supplemental_Files [file gkaf1007_supplemental_files.zip › SupplementaryFigure2.pdf]

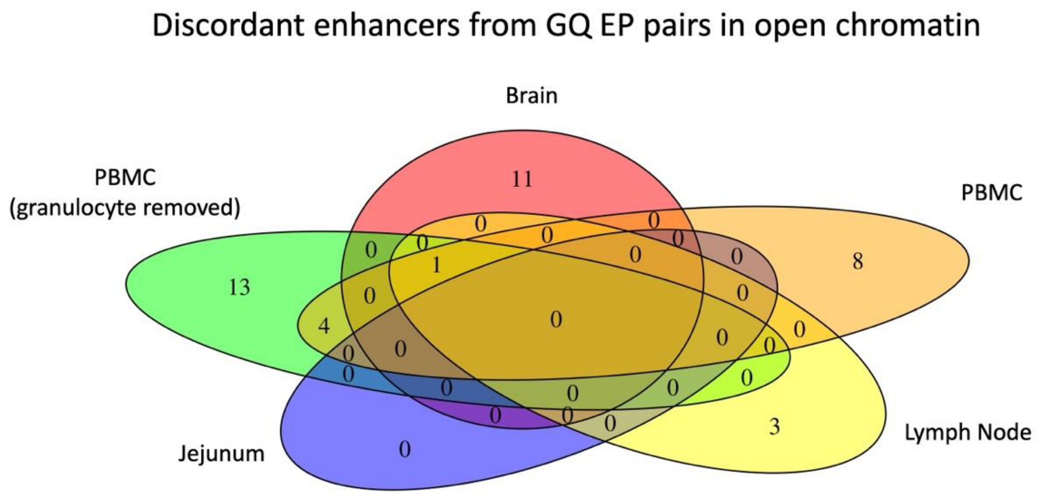

Supplementary Figure 2. Number of discordant enhancers from GQ pEP pairs in open chromatin based on sCell Multiome ATAC + Gene Expression Data.
